# Microstructural and Mechanical Characterization of Additive Friction Stir-Deposition of Aluminum Alloy 5083 Effect of Lubrication on Material Anisotropy

**DOI:** 10.3390/ma14216732

**Published:** 2021-11-08

**Authors:** Brandon J. Phillips, C. Jacob Williamson, Ryan P. Kinser, J. Brian Jordon, Kevin J. Doherty, Paul G. Allison

**Affiliations:** 1 Department of Mechanical Engineering, University of Alabama, Tuscaloosa, AL 35487, USA; bjphillips1@crimson.ua.edu (B.J.P.); cjwilliamson@crimson.ua.edu (C.J.W.); rpkinser@crimson.ua.edu (R.P.K.); bjordon@eng.ua.edu (J.B.J.); 2 USA Army CCDC, Army Research Laboratory, Aberdeen Proving Ground, Aberdeen, MD 21005, USA; kevin.j.doherty18.civ@mail.mil

**Keywords:** additive friction stir-deposition, additive manufacturing, aluminum alloy, defects, solid-state, fractography, tensile, electron backscatter diffraction, microhardness

## Abstract

Additive Friction Stir-Deposition (AFS-D) is a transformative, metallic additive manufacturing (AM) process capable of producing near-net shape components with a wide variety of material systems. The solid-state nature of the process permits many of these materials to be successfully deposited without the deleterious phase and thermally activated defects commonly observed in other metallic AM technologies. This work is the first to investigate the as-deposited microstructure and mechanical performance of a free-standing AA5083 deposition. An initial process parameterization was conducted to down-select optimal parameters for a large deposition to examine build direction properties. Microscopy revealed that constitutive particles were dispersed evenly throughout the matrix when compared to the rolled feedstock. Electron backscatter diffraction revealed a significant grain refinement from the inherent dynamic recrystallization from the AFS-D process. Tensile experiments determined a drop in yield strength, but an improvement in tensile strength in the longitudinal direction. However, a substantial reduction in tensile strength was observed in the build direction of the structure. Subsequent fractographic analysis revealed that the recommended lubrication applied to the feedstock rods, necessary for successful depositions via AFS-D, was ineffectively dispersed into the structure. As a result, lubrication contamination became entrapped at layer boundaries, preventing adequate bonding between layers.

## 1. Introduction

Metallic additive manufacturing (AM) is a rapidly growing field involving a multitude of processes for an even larger number of metallic materials supporting a variety of end uses [1,2,3]. However, each process has its own inherent benefits and shortcomings that are dependent on both the selected materials and geometrical constraints. Similar to laser welding, selective laser sintering/melting (SLS, SLM) directs a focused, high-energy laser at a bed of powdered material to create a melt pool that fuses nearby powder together, layer-by-layer, until the desired geometry is achieved. However, the high thermal gradients pose issues for conductive, low melting temperature materials such as aluminum alloys, such as porosity [4], hot tearing [5], and vaporization of alloying elements such as magnesium [6]. As a result, relatively little work is conducted on commercial aluminum alloys within the realm of fusion-based AM techniques and often requires the use of elemental additives or atomized casting alloys [7,8].

To combat selectively vaporizing magnesium in Al-Mg systems, wire-arc additive manufacturing (WAAM) employs a GMAW style welding system in tandem with CAM software to deposit spooled wires of aluminum into 3-D parts [9,10]. Horgar et al. [11] produced an AA5183 WAAM deposit to evaluate the intrinsic microstructural and mechanical properties resulting in the Al-Mg alloy. The study found that the intense thermal cycling of the fusion-based process produced voids and hot cracks developing along grain boundaries. Alleviating some of the porosity, Fang et al. [12] altered the arc pulse of their commercial WAAM system to relegate the energy input during deposition. By adjusting the energy input frequency, a lower, yet more stable thermal profile was achieved, which reduced but did not eliminate the area fraction of pores observed in the final product. However, the thermal profiles still promoted dendritic grain structures along the build direction. Similarly chemical analysis following directed energy deposition (DED) of AA5083 by Svetlizky et al. [13] demonstrated that the as-deposited material composition more closely matched AA5754 due to the evaporation of Mg and Zn.

Interestingly, an alternative to the fusion-based manufacturing processes, solid-state processes exert significant stresses on feedstocks to induce severe plastic deformation to promote diffusion between interacting materials while remaining below material melting temperatures. Thus, solid-state processes generally avoid phase transformation and thermally activated defects [14]. Ajdelsztajn et al. [15] performed some of the first work of cold gas dynamic spray (CS) of AA5083 by using commercially obtainable atomized and cryomilled powder. The work established the forces of the particles impacting the workpiece dynamically recrystallized grains into the nanocrystalline regime, which provided a Vicker’s hardness of 261 Hv. The study additionally established that by providing a large, irregular particle surface, better mechanical interlocking was possible between deposited particles, virtually eliminating porosity caused by poor adhesion. Rokni et al. [16] obtained similar results in CS AA5083, but observed that deposited grains appeared dislocation free. The authors note that the stark increase in hardness was attributed to the nanocrystalline grains providing Hall-Petch strengthening and particles resulting from the induced plasticity, which was observed through transmission electron microscopy.

**Figure 1:**
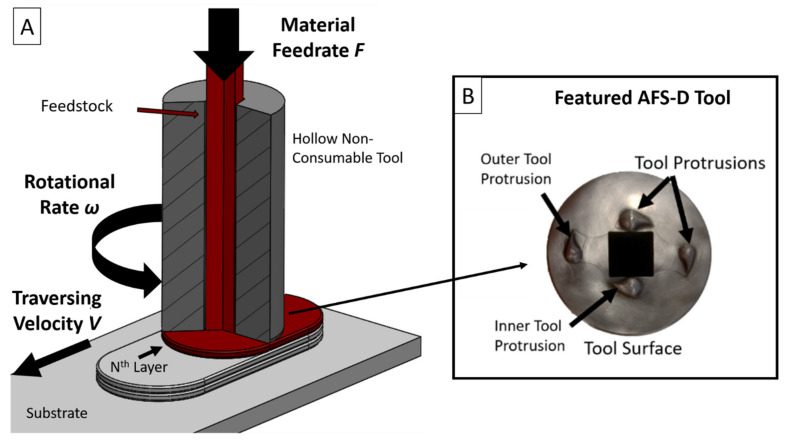
(**A**). Schematic of the AFS-D process denoting specific features and process control variables. (**B**) Schematic of the features located on the interaction face of the steel AFS-D tool.

More recently, the solid-state Additive Friction Stir-Deposition (AFS-D) process has been introduced as a low energy, high deposition rate AM technique to deposit a wide range of alloys [17,18,19,20,21]. AFS-D utilizes a non-consumable rotating tool that exploits frictional heat and intense shear stresses to plasticize material as it traverses along a substrate. The center of the tool extrudes solid or discontinuous feedstock at a defined feed rate as the tool traverses at a specified layer height above the substrate or prior layer, shown schematically in Figure 1A. At the end of a layer path, the tool raises to the proceeding layer height to create multiple layers of material. The AFS-D process has already been demonstrated to provide paths to additively manufacture [22], coat [23], repair [24], and recycle [25] materials in a single AM process. Processing temperatures in AFS-D have been reported by Garcia et al. [26] to be near the solidus temperature of deposited Al-Mg-Si. While depositions were possible, congruent work on Al-Mg-Si by Rutherford et al. [27] and Phillips et al. [28] found that the temperatures required to produce these deposits eliminated the heat treatment of the T6 tempered feedstock by dissolving β″-phases back into the matrix. Investigations on Al-Zn-Mg-Cu by Avery et al. [29] observed similar behavior where preferential η-phase precipitates were redistributed. The conclusions from each of these studies determined that the significant processing temperatures dissolved the traditional strengthening precipitates in the respective alloys; thus, providing a weaker tensile and microhardness response. In work on the AFSD of Titanium alloy Ti64, Agrawal et al. [30] determined that the stable α phase was dissolved and subsequently precipitated during the deposition process due to the temperature of the material exceeding the beta transus temperature with the material resultingly demonstrating an increase in initial yield and ultimate strengths. Similarly, when investigating solid-solution strengthened materials such as Inconel 625, both Rivera et al. [31] and Avery et al. [17] observed improved mechanical behavior due to significant grain refinement, equiaxed morphologies, and distribution of constituent phases throughout the as-deposited material. Of interest in the AFSD deposition of aluminum, tool wear has been identified in harder alloys such as titanium [30] and Inconel [17] to occur directly along the layer interface, but in softer alloys such as aluminum [18,32,33] and copper [34] there has not been any evidence of tool wear. The decreased wear from these softer alloys is likely a combination of the effect of lower stress on the tool as well as the lower operating temperatures as the deposition temperature has been seen to be a function of the melting point of the feedstock material.

AFS-D is a derivative of friction stir welding and processing (FSW/P) in which frictional heat and high shear forces mechanically and metallurgically join one or more materials by way of a non-consumable shoulder and pin tool. Owing to similar physics, many similarities can be drawn. In FSW of AA5083, investigations by Mishra et al. [35] and Lombard et al. [36,37] determined that processing parameters significantly altered the microstructure, mechanical performance, and size of the processed zone. Particularly, two dominant parameters were determined to affect each of these features: tool rotational rates, *ω,* and tool traversing speed, *V*. These values were then correlated to mechanical performance in numerous aluminum alloys by Balasubramanian [38] through the introduction of weld pitch, originally denoted as, *V*/*ω* [39,40]. In many of these studies investigating this ratio, it is seen that rotational speed has a stronger influence on temperature generation in the workpiece; but can be carefully controlled by adjusting the travel speed to prevent thermal degradation in AA5083 components [41,42]. FSW effects on strength of Al-Mg alloys have been investigated previously with Kuryntsev et al. observing a strengthening of the weld nugget as compared to the parent material in a binary alloy AlMg5 [43]. Bodukuri et al. compared the tensile strength of friction stir welded and tungsten inert gas welded AA5083 specimen and demonstrated the FSW specimen have tensile strengths vastly higher than the solidification-based weld [44].

Since weld pitch has been designated as a governing factor to achieving greater as-deposited material strength, this work will evaluate processing rates by using weld pitch as a relationship to produce the first substantial deposits of solid-solution strengthened AA5083. However, as an additive process, the material feed rate, *F*, must also be considered when investigating processing conditions. Like weld pitch, the feed rate can be related to the tool traversing velocity in a similar ratio defined as the deposition ratio, *A*/*V*, which describes the quantity of material deposited per unit distance traveled. With tool velocity as a constant, we can focus on the rotational speed and material feed rate as two variable parameters for this work. This investigation, for the first time, evaluates the as-deposited microstructure before evaluating bi-directional mechanical behavior of a AA5083 free-standing deposit produced via AFS-D.

## 2. Materials and Methods

A B8 MELD machine affixed with a 4-teardrop hardened steel tool (Figure 1B) was employed to produce the deposits evaluated in this work. Feedstock material utilized in this study was wire-EDM cut into 9.53 mm× 9.53 mm× 254 mm rods parallel to the rolling direction from a 63.5 mm thick AA5083-H131 plate. The as-machined feedstock was then coated with a high-temperature graphite aerosol lubricant as instructed by the machine manufacturer to prevent friction within the walls of the AFS-D tool while depositing material. The lubricant is established as a necessary part of the process to impede the tool from jamming during nearly all aluminum deposits. A 9-sample processing window, tabulated in Table 1, was produced altering the tool rotational speed from 200–400 RPM and linear actuator feed rate, expressed as volumetric feed rate, from 6341–8664 mm^3^/min. Tool traversing velocity was maintained at 127 mm/min based off of prior aluminum alloy parameterization work by Phillips et al. [28], and to reduce the processing window size to evaluate the individual effects of weld pitch, expressed as *V*/*ω* by FSW, and deposition ratio, which is defined as *A*/*V* where A is the linear actuator velocity. To provide an estimate on processing temperatures, a thermocouple was placed in a pre-drilled hole half the thickness of the substate while a second thermocouple measured the ambient temperature. Deposits for the parameter study were 50.8 mm center-to-center and 4.064 mm tall. The follow-on larger free-standing deposit was manufactured to be 228.6 mm center-to-center in length and 66.04 mm in height to permit the full geometry of the tensile samples oriented in the build direction. A single non-consumable AFS-D steel tool with 4 teardrop features was employed to deposit all 10 builds evaluated. As the machine used for deposition did not have a continuous feed system, the deposition of the larger deposition was occasionally between layers to allow the insertion of additional feedstock material into the opening of the tool. For the 9-sample processing window, the depositions were able to be continuously deposited without the need for additional feedstock material due to their smaller size.

Samples produced in the parameter study were cross-sectioned, ground stepwise down to a 4000p grinding disc, and polished with a 3 µm water-based diamond suspension. Samples were then tested on a Clemex CMT microindenter (Clemex, Longueuil, QC, Canada) with 16 × 45 grid, 500 µm spacing, and 200 gf. Microscopy samples from the cross-sections were then polished further down with a 0.03 µm colloidal silica solution. Optical microscopy (OM) was performed on a Keyence VHX-7000 digital microscope (Keyence, Osaka, Japan). Scanning electron microscopy (SEM) was conducted on a Tescan Lyra FE-SEM (Tescan, Brno, Czech Republic) with an Octane Elite EDS system and EDAX Hikari Super EBSD camera to perform chemical, grain morphological, and fractographic analysis. EBSD and fractographic analysis was performed at 20 keV while EDS was performed at 5–15 keV to evaluate lower energy elements within the deposits. Monotonic tensile tests were conducted on a MTS Landmark servo hydraulic load frame (MTS, Eden Prairie, MN, USA) with a 25 kN load cell in ambient lab temperature and humidity. Three specimens were prepared from the same wrought AA5083-H131 plate as the feedstock parallel to the rolling direction using a wire-EDM. As-deposited AFS-D AA5083 samples were prepared in the same manner in the longitudinal and build directions to evaluate any anisotropy. The experiments were run in displacement control with a 5 mm gage extensometer. The tests were conducted in the quasi-static regime with a displacement rate of 0.005 mm/s leading to a strain rate of 0.001 mm/mm/s across the gage section.

## 3. Results

### 3.1. Parameter Study

**Figure 2:**
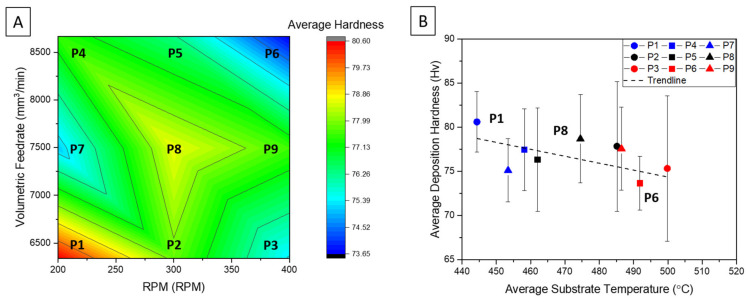
(**A**). Color contour plot contrasting the material volumetric feed rate to the RPM of the tool. (**B**) Deposition cross-sectional hardness versus average temperature. Key: circles correlate to 200 RPM, squares to 300 RPM, and triangles to 400 RPM. Blue data points correlate to deposition ratio of 0.55, black data points correlate to a deposition ratio of 0.65, and red data points correlate to a deposition ratio of 0.75.

Figure 2A illustrates the correlation between the volumetric input of material into the deposit versus the AFS-D tool rotational speed with respect to the average Vicker’s hardness of the cross-section of each parameter set evaluated. The contour plot reveals, that maintaining a lower RPM and minimizing material input proved to retain 80% of the wrought AA5083-H131 feedstock hardness measured at 102 Hv. Additionally, the value of P1 lies between the Vicker’s hardness of friction stir welds in the nugget and thermo-mechanically affected zone in AA5083 reported by various studies [42,45,46]. In these published works, processing speeds played a significant role in the severity and variation of the reduction in hardness where increases in rotational speed or a decrease in tool translational speed correlated to a lower weld pitch. The effect of low weld pitch is observed in the published literature as well as evident by the reduced hardness evident in P6.

Figure 2B further evaluates this effect, which plots the deposition hardness from Figure 2A against the temperatures observed by the substrate-embedded thermocouples. In Figure 2B, circular symbols correlate to the low rotational rate conditions (200RPM), squares indicate the medium rate (300 RPM), and triangles represent the highest rotational rates evaluated (400RPM). Volumetric feed rates are classified by color where the minimal material input is denoted in blue, the median rate in black, and the highest feed rates in red. Figure 2B further corroborates that an increase in RPM induces greater heat into the deposit while maintaining a constant tool traversing speed. Thus, similar relationships can be drawn in AFS-D to FSW where weld pitch has a significant effect on mechanical properties when processing aluminum alloys, regardless of precipitate or work-hardening strengthening mechanisms. It is also notable that increases to material feed rate plays a role in the increase in deposition temperature. This effect was evaluated by Garcia et al. [26] in Cu and Al-Mg-Si resulting from the partial slipping-sticking state of the actively deposited aluminum’s interaction to the AFS-D tool face and features. The study notes that the friction coefficient between aluminum and steel is significant, which leads to greater material sticking between the deposited material and tool surface. In this work, greater material flow correlates to greater contact between the deposition layer and tool; and thus, an increase in temperature. From a macroscopic viewpoint, deposits that exhibited greater temperatures also presented rougher surface finishes, and in some cases, significant aluminum sticking to the tool causing some of these surface defects. To maintain the highest mechanically sound deposition based on the parameter study, P1 was determined to be the optimal parameter to be evaluated and employed in the subsequent, large-scale deposit at a Vicker’s hardness of 82.9 ± 4.7 Hv.

**Figure 3:**
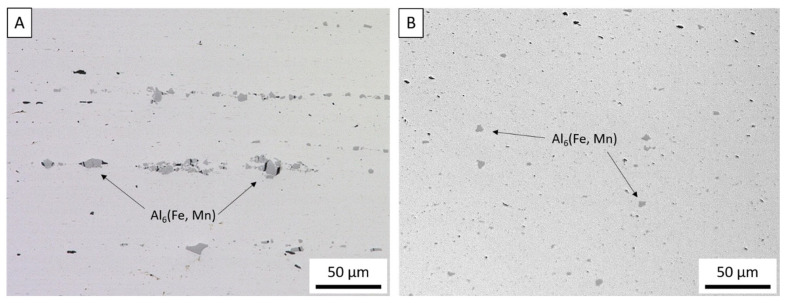
(**A**). Optical micrograph of wrought AA5083-H131 plate showing constitutive particles pancaked between the rolled grains. (**B**) Optical micrograph of as-deposited AA5083 after AFS-D showing smaller, dispersed constitutive particles.

Optical micrographs of the wrought AA5083-H131 feedstock and as-deposited AA5083 are depicted in Figure 3A,B, respectively. Despite surface defects caused by less desirable process parameters, the AFS-D process fills in surface defects of the previous layers on the subsequent layers. As such, no volumetric defects were observed in the 9 cross-sections from the preliminary parameter evaluation. The size and distribution analysis of these particles is summarized in Table 2. The authors note that no statistical analysis was conducted delineating the Fe or Mn particles from the possible formation of β-phases. The feedstock exhibits typical rolled microstructural features where large constitutive particles are pancaked parallel to the rolling direction of the wrought AA5083-H131 control, which are observed as the dark particles in the figures. The result of the rolling process effectively creates stringers and layers of aluminum lined with these elongated Al_6_Fe and Al_6_Mn secondary phase particles. Once processed via AFS-D, these 6.7 µm particles are fractured to nearly half their original size at 3.8 µm. The secondary phase is then dispersed homogenously throughout the microstructure due to the high levels of shear-induced plasticity from the process as evident by the lower deviation in nearest neighbor distance.

**Figure 4:**
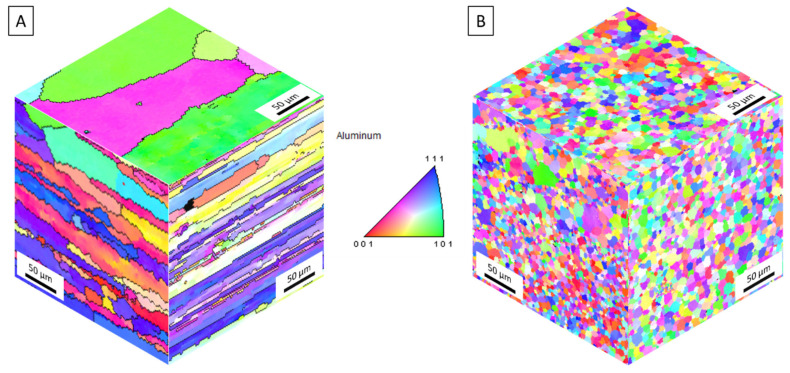
EBSD maps in 3-orientations of (**A**) the rolled AA5083-H131 feedstock and (**B**) as-deposited AA5083. Scale bars are 50 μm.

Figure 4A,B depict inverse pole figures displaying the stark reduction in grain size from the AA5083-H131 feedstock and as-deposited AA5083 in the longitudinal/rolling (top), long transverse (right), and short transverse/build (left) directions. The feedstock exhibits typical rolled grains along the longitudinal direction on the order of 250 µm with thin pancaked grains along the transverse directions. Following AFS-D, these grains are refined into 3.16 µm diameter equiaxed grains with a standard deviation of 1.38 µm. As denoted by recent studies of Al-Cu [18], Al-Mg-Si [27,28,47], and Al-Zn Mg-Cu [29,48], AFS-D refines aluminum alloy grain structures via geometric, continuous dynamic recrystallization. Mason et al. [33] established that grain sizes were generally larger in the first layers of the deposit due to the increased thermal exposure as additional layers are built. In this work, AFS-D AA5083 exhibited no noticeable difference in grain size, particularly through the gage section as seen in Figure 4B.

### 3.2. Mechanical Behavior of the Free-Standing Deposit

**Figure 5:**
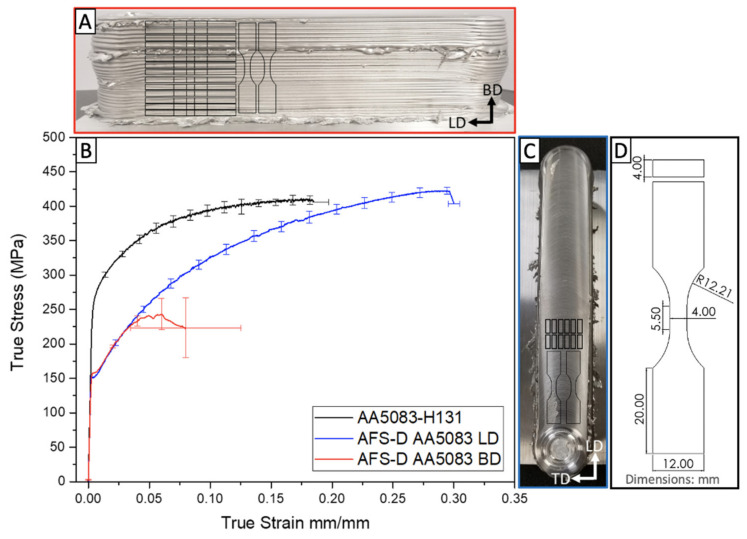
(**A**). Profile view of AFS-D AA5083 deposit. (**B**) Comparative tensile stress-strain response of the wrought AA5083-H131 feedstock and as-deposited AA5083. (**C**) Top view of AFS-D AA5083 deposit. (**D**). Sample geometry used in this study.

Sample geometry, locations, and the evaluated bi-directional monotonic tensile properties of the as-deposited AFS-D AA5083 versus the properties of the feedstock AA5083-H131 is compared in Figure 5A–D. Additionally, the experimental data is available in Table 3. Two longitudinal (LD) samples from each: the first few layers, middle tier layers, and final layers of the deposit shown in Figure 5A were evaluated to establish strength of the deposited material. However, due to the abnormal mechanical response in the build direction (BD), ten samples were mechanically tested to determine the cause of the anisotropic behavior. Samples tested in both the LD and BD exhibited a severe reduction in yield strength resulting from the AFS-D process. The thermo-mechanical processing shears grains through lattice rotation and annihilates dislocation structures. As a result, the refined grains lose their dislocations within the grains; and thus, any work-hardening effects introduced by the H131 treatment. However, it is noted that beyond the yield point, the as-deposited material begins to strain-harden with increasing strain. In the longitudinal sample, we find that the ultimate tensile strength exceeds that of the feedstock AA5083-H131 material by approximately 20 MPa. This increase can be directly attributed to the reduction of grain size down to 3.1 µm, which the Hall-Petch relationship correlates to a 24.6 MPa increase in strength. The build direction, however, reveals a significant reduction in strength and ductility unlike any currently reported data on AFS-D aluminums. Premature fracture occurring in the plastic regime of the material’s response suggests that significant defects are present as a result of the layer-by-layer process.

**Figure 6:**
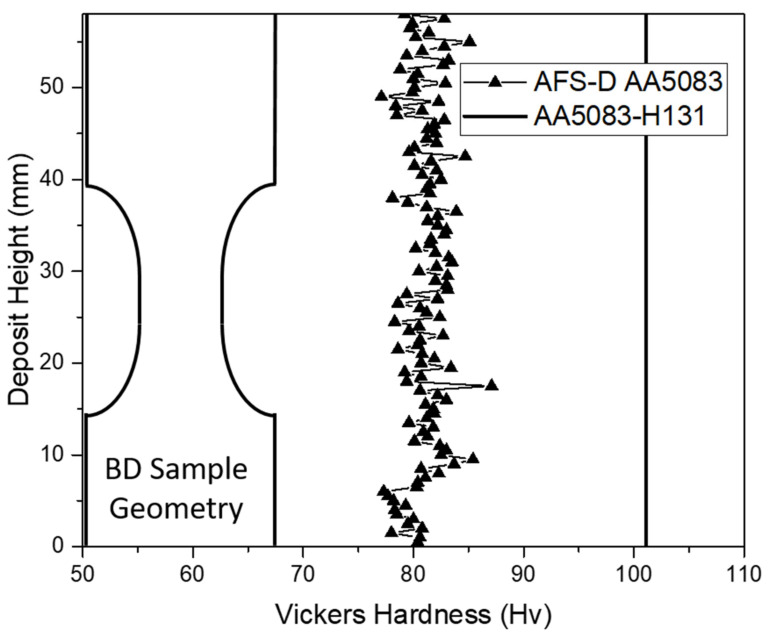
Vicker’s hardness plot with respect to the sample geometry for both AA5083-H131 control and AA5083 processed with AFS-D.

To determine the presence of a strength gradient in the build direction of the free-standing deposit, a section was extracted from the cross-section and indented as examined in Figure 6. The hardness plot compares the as-deposited AFS-D AA5083 hardness to the measured feedstock values. As displayed, the recorded Vicker’s hardness values are aligned to those previously evaluated in the parameter study at 81.1 Hv. The consistency between parameter set P1 and the hardness data from the cross-section of the larger deposit deduce that the additional heat and plastic deformation induced as a result of the additional layers neither strengthens nor weakens the work-hardened AA5083. Additionally, no significant reduction in strength is exhibited in the gage section, which suggests that microstructural defects may exist between layers of the AFS-D AA5083.

### 3.3. Post-Mortem Analysis

**Figure 7:**
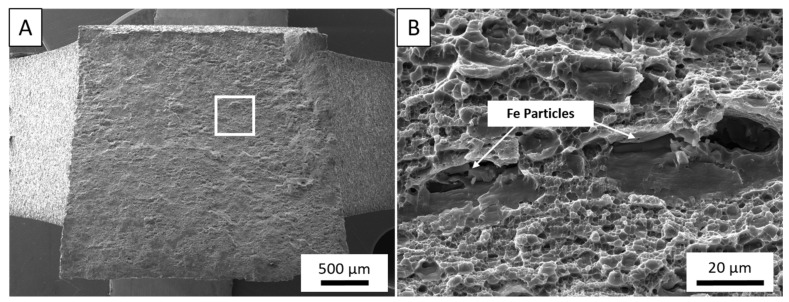
(**A**). Low magnification overview of 45° fracture of a AA5083-H131 control sample. (**B**) Large voids, surrounded by microvoids, coalesced from fractured iron particles along rolling boundaries.

To evaluate and contrast the failure mechanisms between the feedstock material and as-deposited AA5083, Figure 7A,B present fractographic evaluation of the control AA5083-H131 tested in the rolling direction of the plate. The tensile loading produced a fracture plane 45° with respect to the loading direction. The entirety of the fractured surface is dominated by microdimpling surrounding larger voids originating from brittle, cracked iron-rich inclusions such as those displayed in Figure 7B. The smaller dimples are attributed to localized plasticity reaching critical strain while voids coalescing from constitutive particles develop until transgranular fracture occurs as is typical for wrought AA5083-H131 [49].

**Figure 8:**
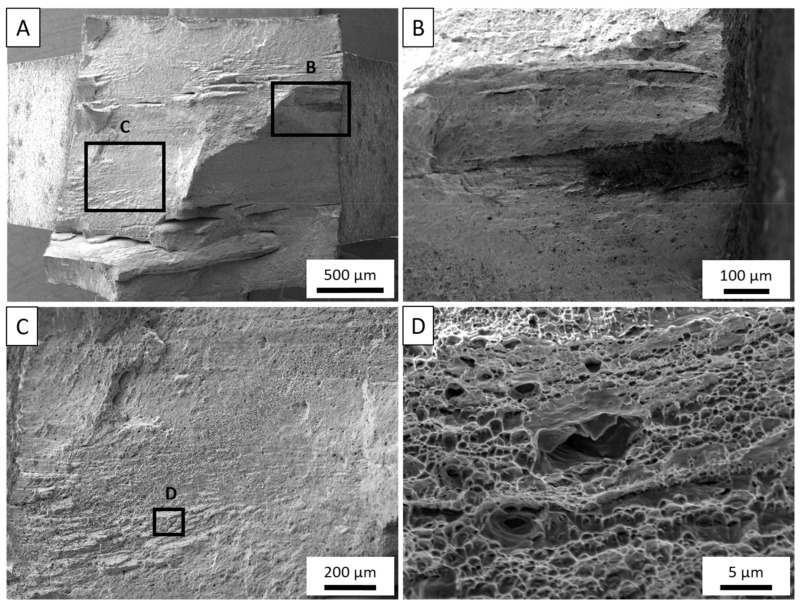
(**A**). Post-mortem analysis of tensile sample tested in the horizontal, longitudinal direction. (**B**) Contamination observed near delamination crack. (**C**) Material flow lines parallel the deposited layers with a larger void encasing a secondary-phase particle. (**D**) Traditional ductile dimpling and larger void encasing a secondary-phase particle observed.

Figure 8A–D depicts a fractured sample tested from the longitudinal direction of the free-standing deposit. A few features are immediately evident in the macroscopic view in Figure 8A. First, cracks appear to be forming approximately 1 mm apart, which is the approximate layer height for each layer in the deposit. Towards the center of the sample, the fracture comes to a point and gradually slopes to the exterior edges of the sample. These paths closely match the rotational pattern of the tool features as the tool traverses in the direction, observed collinearly with the loading direction of this sample. Next, highlighted in Figure 8B, a dark, discolored region is observed at a large crack originating from the exterior surface of the sample. In this region, dimpling appears very shallow as the dark particles fill the dimples. Further discussion of this region is presented later. Between the layer boundaries, material flow lines are observed in Figure 8C and at higher magnification in Figure 8D. This region experiences more traditional fracture where transgranular dimpling surrounds larger voids nucleating from constitutive particles. However, it is observed that rather than uniquely Al_6_ (Fe, Mn) particles, these platelets more closely resemble that of the β-phase precipitate. Yan et al. [50] found that β would often form as a result of dislocations diffusing Mg atoms towards high-angle grain boundaries (HAGB), particularly in the presence of Al_6_Mn during deformation processes. In AFS-D of aluminum alloys, it has been noted through various works that the high shear mechanisms of the process typically promote a high density of HAGB proportional to the strain governed by processing conditions [28,29,47]. As a result, the constitutive particles located at grain boundaries also provide preferential locations to precipitate β-phases. In loading conditions, larger voids can be expected to develop as a result of both Al_6_ (Fe, Mn) and Al_3_Mg_2_ particles.

**Figure 9:**
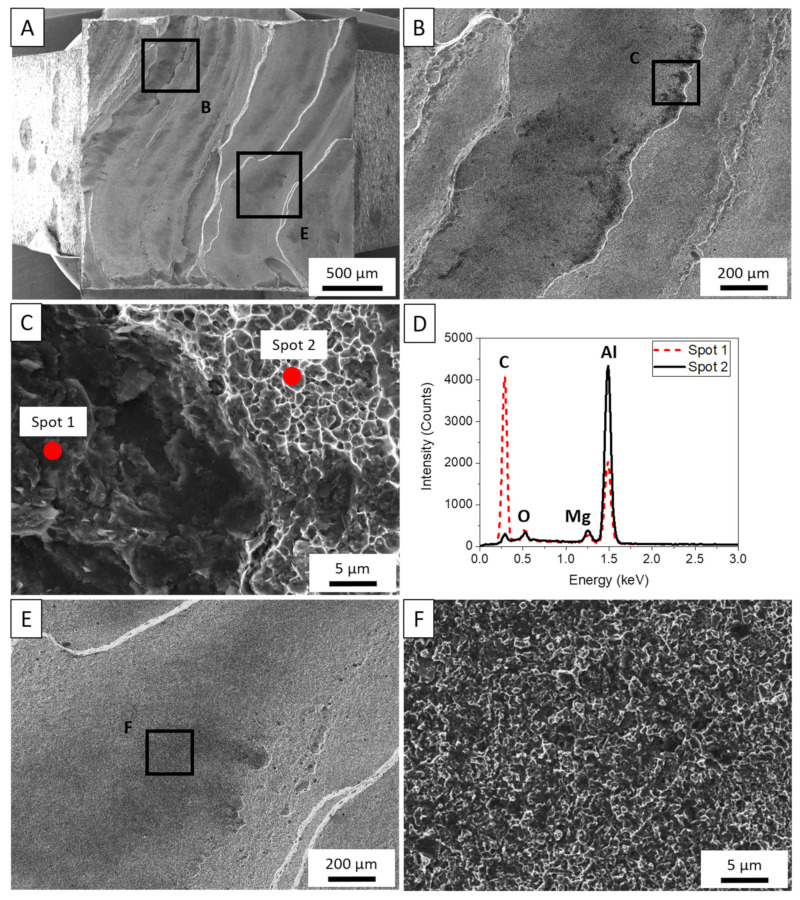
(**A**). Post-mortem analysis of tensile sample tested in the vertical build direction. (**B**) Contamination evidenced along the fracture surface. (**C**) High-magnification image of two locations compared via EDS. (**D**) EDS analysis comparing contaminant composition with respect to the matrix. EDS was performed at 5 keV. (**E**) Brittle fracture features observed. (**F**) Shallow dimples are filled with carbon particles.

Figure 9A–F collates fractographic and EDS analysis of a sample tested in the build direction. The BD samples reveal a flat fracture surface, characteristic of a brittle failure, with tearing ridges separating thin layers of material. Near some ridges, the dark particles are observed in greater quantities such as those seen in Figure 9B. Two regions were probed to determine the composition of the contaminants in Figure 9C,D. As previously observed in the fractographic analysis of the LD samples, these dark regions are confirmed to be carbon contamination from the lubrication. Figure 9E,F show that even away from the ridges, the carbon particles sit within shallow dimples throughout the surface of the build direction samples.

Until the present work, it was surmised that the carbon-based lubricant, employed to prevent sliding friction in the confines of the tool walls, were either burnt off during deposition or sufficiently mixed into the deposit by the repetitive stirring motion. Instead, it appears that this carbon displaces to the layer boundary and is only moderately coerced into the layer itself by the tool features on the subsequent passes as observed in Figure 8. The results of this analysis show that carbon does not distribute deep into layers, but rather remains on or near the surface of each layer produced. Moreover, the carbon does not diffuse into the matrix well, but rather becomes entrapped due to their incoherency with the surrounding matrix. The entrapped carbon prevents adequate material diffusion between layers; thus, creating a large interlayer defect and subsequent brittle behavior as evidenced in this work. The inhomogeneity of carbon content in the deposition is demonstrated in the cracking seen in the horizontal specimen, demonstrated in Figure 8. The presence of the distinct bands of cracking that can be seen to correlate to layer height clearly illustrates that a brittle intermetallic is being formed in distinct bands and not distributed throughout the layer as previously thought.

**Figure 10:**
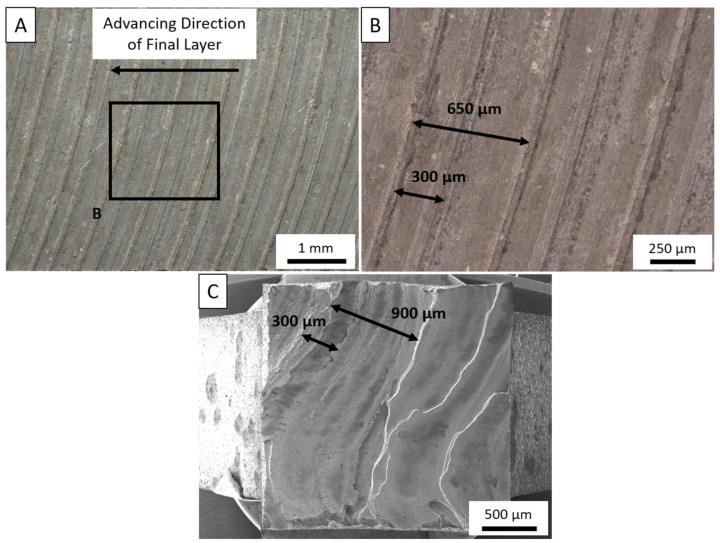
(**A**). Macroscopic image of the onion ring structure on the final layer of the deposit taken as observed directly below the tensile sample observed in (**C**). (**B**) High-magnification OM of the onion ring pattern depicting distances between rings. (**C**) SEM fractography correlating flow patterns with onion ring structures observed in OM.

The spacing of these ridges in the BD fractography provide insight into the carbon contamination, which is correlated in Figure 10A–C. As is traditional in friction stir-based processes, an onion skin structure is observed on the surface of the deposit. As per Perry et al. [51], extraneous, unconstrained material flow develops a wave behind the tool generating a rough semicircular pattern. At tool movement speeds of 200 RPM and 127 mm/min, the spacing of these rings are 609 µm, which closely resembles the spacing measured on the top surface of the deposit where variance can be directly attributed to machine compliance. However, between each ring, a shallower second ring is developed approximately 300 µm from the leading onion ring. This second ring is excess material flowing from the edge of each individual ring, typically denoted as flash. Evaluating the spacing of the tearing ridges from the BD samples and the surface of the deposit, the features of the fracture surface match those of the onion rings.

**Figure 11:**
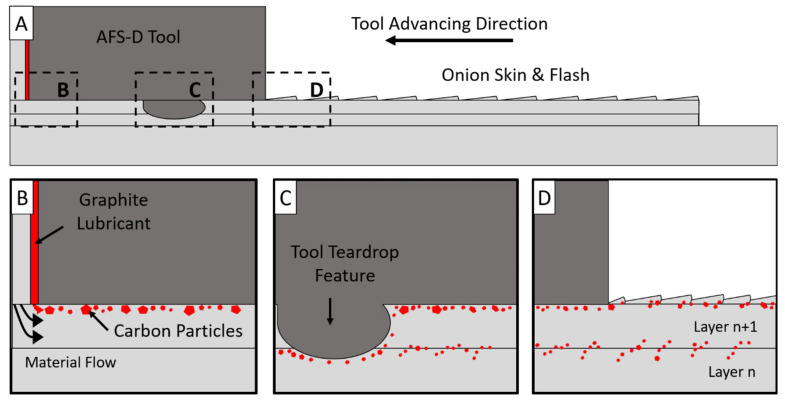
(**A**). Overview schematic of the cross section of the AFS-D tool, feedstock, and two layers. (**B**) Schematic of the material flow close to the tool exit hole. Carbon highlighted in red. (**C**) Schematic of the area near a tool teardrop feature. Carbon follows the path of the tool features rather than dissolving into the layers. (**D**) Carbon on the active layer becoming entrapped by the flash from excess material in the onion skin.

The observations of both the fractography and top surface of the deposit paints a picture of the following phenomena hypothesized and graphically shown in Figure 11A–D. First, as material is deposited, the material flows like a viscous fluid radially from the exit hole of the tool. However, since material is constrained, material flow is prohibited from mixing well in the vertical direction. This means that material towards the outer perimeter of the feedstock becomes compressed between the material ahead of the presently deposited layer, below the layer, and the tool interaction face. More specifically, the outer layer of carbon, which is located on the surface of the feedstock, becomes compressed and flows along the top surface of each layer as the layer is deposited (Figure 11B). Additionally, as the previously mentioned unconstrained wave of material develops the onion ring structure, it encases the now trapped carbon particles along the layer boundary in the 300 µm wave of flash, schematically shown in Figure 11D. The theory behind Figure 11B is supported by similar techniques employed in FSP to generate surface composites in which powders or solutions are applied to the surface of a workpiece and subsequently mixed in via FSP [52]. However, rather than an existing solute on the surface of the substrate, AFS-D introduces foreign material from the feedstock coating. In FSW, this phenomenon may be changed by the effect of tool tilt. However, in this work on AFS-D AA5083, no tool tilt was applied during the production of these depositions.

It is worth noting that tool features should disrupt and distribute the layer of carbon particles on the subsequent passes, particularly considering the central region of the deposit is stirred four-times per rotation. While true, the fractographic images of the LD from Figure 8 show that the features merely push the carbon particles into the grooves cut by the features and is shown schematically in Figure 11C. The stirring dynamics invoked by the tool features provide some distribution of carbon away from the layer boundaries, but the stirring motion is insufficient enough to completely disrupt the layer interfaces, at least at 200 RPM. This concept originates from FSW studies in which a contrasting marker material was placed into the workpiece prior to FSW before non-destructive evaluation. In AA2024, Schmidt et al. [53] used a copper strip to visualize the flow using X ray computer tomography to highlight the flow of the copper within the weld. The authors note that some of the copper is pulled downward into the cavity produced by the FSW pin. However, at higher locations in the weld nugget, material rotates with the tool and remains relatively unchanged with respect to vertical location in the weld, similar to the findings of this study. To properly evaluate this phenomenon, the authors suggest an in-depth investigation on the effect of tool geometries on the mechanical stirring of the AFS-D process as well as their influence on necessary lubrication.

## 4. Conclusions

This investigation is the first work to evaluate the microstructural and mechanical behavior of as-deposited AFS-D AA5083. The study reached the following conclusions:

In processing AA5083 by AFS-D, greater mechanical strength was expected with lower tool rotational rates due to less heat generation in the deposit as determined by microindentation experiments covering the cross-section of 9 deposits of varying processing conditions.

1. Equiaxed grains resulted from dynamic recrystallization and a 97% reduction in grain size was exhibited with an average grain size of 3.16 µm. While the thermal processing reduced the yield strength due to annihilation of dislocations, the ultimate tensile strength was improved by 5.2% due to the grain size reduction.
2. Build direction properties were greatly depreciated and macroscopic brittle fracture was observed with a 73.3% reduction in strain to failure and a 42.9% reduction in ultimate tensile strength.
3. The cause of premature failure in the build direction was attributed to the influence of carbon contamination preventing diffusion of the matrix between layers.
4. Carbon contamination originates from the aerosol lubricant, used to prevent friction within the tool during deposition, that becomes entrapped by the flash and material from the consecutive layers. Elimination of this contamination could provide consistency between the longitudinal and build directional mechanical performance.
5. As an immature process, more work is suggested to investigate the influence of tool geometry and design to assist in mitigating interlayer defects. Additional work should also include evaluation of alternatives to carbon-based lubrication techniques.

## Figures and Tables

**Table 1:** Sample set nomenclature and parameter specifications.

Parameter Set	Rotational Speed [RPM]	Vol. Feed Rate [mm^3^/min]	Weld Pitch [mm/rot]	Deposition Ratio
P1	200	6341	0.635	0.55
P2	300	6341	0.423	0.55
P3	400	6341	0.318	0.55
P4	200	8664	0.635	0.75
P5	300	8664	0.423	0.75
P6	400	8664	0.318	0.75
P7	200	7494	0.635	0.65
P8	300	7494	0.423	0.65
P9	400	7494	0.318	0.65

**Table 2:** Particle analysis comparing constitutive particles in the AA5083-H131 feedstock and AFS-D AA5083.

Sample	Particle Size			Nearest Neighbor Distance		
	Max	Average	Standard Deviation	Max	Average	Standard Deviation
	µm^2^	µm^2^	µm^2^	µm	µm	µm
AA5083-H131	64.6	6.86	10.86	75.6	10.46	8.39
AFS-D AA5083	56.3	3.80	6.13	42.2	8.30	5.99

**Table 3:** Summary of AA5083-H131 feedstock and as-deposited AFS-D AA5083.

Material (Direction)	E (GPa)	σ_YS_ (MPa)	σ_UTS_ (MPa)	ε_f_
AA5083-H131	82.9 ± 0.9	273.7 ± 1.0	410 ± 6.1	0.15 ± 0.024
AFS-D AA5083 (LD)	70.8 ± 5.2	151.3 ± 1.7	431.3 ± 1.9	0.30 ± 0.005
AFS-D AA5083 (BD)	68.9 ± 5.8	157.7 ± 1.2	246.2 ± 45.9	0.08 ± 0.045

## Data Availability

Data presented in this study is available on request from the corresponding author. Data is not publicly available due to ongoing, concurrent efforts in this subject.
